# The population randomization observation process (PROP) assessment method: using systematic habitation observations of street segments to establish household-level epidemiologic population samples

**DOI:** 10.1186/s12942-019-0190-z

**Published:** 2019-11-08

**Authors:** Mieka Smart, Richard Sadler, Alan Harris, Zachary Buchalski, Amber Pearson, C. Debra Furr-Holden

**Affiliations:** 1 Division of Public Health, College of Human Medicine, Michigan State University, 200 E. 1st Street, Flint, MI 48502 USA; 2 Dept of Geography, Environment & Spatial Sciences, College of Social Science, Michigan State University, 673 Auditorium Road, Room 115, East Lansing, MI 48824 USA

**Keywords:** Random sample, Census block group, Habitation, Population sample

## Abstract

**Background:**

Identifying and intervening on health disparities requires representative community public health data. For cities with high vacancy and transient populations, traditional methods of population estimation for refining random samples are not feasible. The aim of this project was to develop a novel method for systematic observations to establish community epidemiologic samples.

**Results:**

We devised a four-step population randomization observation process for Flint, Michigan, USA: (1) Use recent total population data for community areas (i.e., neighborhoods) to establish the proportional sample size for each area, (2) Randomly select street segments of each community area, (3) Deploy raters to conduct observations about habitation for each randomly selected segment, and (4) Complete observations for second and third street segments, depending on vacancy levels. We implemented this systematic observation process on 400 randomly selected street segments. Of these, 130 (32.5%) required assessment of secondary segments due to high vacancy. Among the 130 primary segments, 28 (21.5%) required assessment of tertiary (or more) segments. For 71.5% of the 400 primary street segments, there was consensus among raters on whether the dwelling inhabited or uninhabited.

**Conclusion:**

Houses observed with this method could have easily been considered uninhabited via other methods. This could cause residents of ambiguous dwellings (likely to be the most marginalized residents with highest levels of unmet health needs) to be underrepresented in the resultant sample.

## Background

### Geospatial methods for sampling in population health research

Identifying and intervening on health disparities requires representative community public health data ([19], p. 21–40), often including hard-to-reach subpopulations. Obtaining representative data requires random population sampling methods [6]. In the USA, many random population samples are based on US Census American Community Survey (ACS) data [16]. ACS data are published every year since data collection is an ongoing process, in the form of several useful products [10]. Unfortunately, ACS estimates are susceptible to significantly larger margins of error, compared to the census long-form sample, due to a much smaller sample size [10]. For this reason, in cities with high vacancy and transient populations, traditional methods of population estimation for refining random population sampling frames are not ideal, and some researchers resort to ad hoc combinations of multiple methods (e.g., using secondary data from multiple sources, tracking household visits during survey administration, and soliciting ongoing input from survey administrators) to establish a representative sampling frame [14].

Studies of depopulating neighborhoods have encountered problems with uncertainty about the quantity and distribution of unoccupied homes as well as informal occupation of some “vacant” homes (e.g., squatters) when attempting to establish a reliable sampling frame [14]. Sampson et al. [14] developed a method that accounted for a high number of vacant homes, dynamic depopulation, and officially “unoccupied” residences that were actually inhabited. The authors suggest that researchers working in depopulating areas may benefit from utilizing multiple indicators of occupancy when setting their area frame, in addition to survey administrator input. Further, they recommend that approaches to multiple indicators of occupancy be systematic and iterative for the sake of validity and reliability.

Conducting a population health survey in a rapidly depopulating city and establishing a usable random population sampling frame requires recent population estimates. Moreover, if that city also has a high transiency rate, establishing a usable random population sampling frame requires recent habitation estimates. Stated simply—before going into the field—researchers often want to know how many people to select from each area (population estimate) as well as where people actually live in each area (habitation estimate). This type of design, in which sampling locations are fixed in advance of any data collection, is referred to as a non-adaptive geostatistical design [9]. In a completely random non-adaptive geostatistical design, the sampled locations are comprised of independent random samples from the uniform population distribution in each of the geographic regions of interest [3].

### Other geographic methods for population health research

Sampling methods that are enhanced using Google Earth/Google Street View, remote sensing, and/or drone deployment, are promising, but have limitations as well. Issues include lack of non-visually experienced environmental conditions (smells, sounds, etc.) that might indicate habitation. Other issues include limited camera perspective, lack of recency, and unavailability [15]. Remote sensing techniques for public health were highlighted in the 2014–2015 Ebola virus disease (EVD) epidemic in West Africa. Use of overhead orbital and high-resolution views were instrumental in controversial shaping of attitudes towards specific disease populations and determining response to disease-related threats [5]. Drones make for the dynamic real-time collection of detailed high-resolution data, which can include the collection of non-visual data (e.g., sound, air particulate matter, etc.), but with the potential for drone data collection to be anonymous, issues of privacy are a major concern [7].

There is no existing random non-adaptive geostatistical design method designed specifically for transient and depopulating communities. Such sampling methods could serve useful in nomadic populations, or in cities undergoing significant changes to the structure of settlements (e.g., following a natural disaster). To achieve our objective, we developed a novel process using census data to define neighborhoods combined with direct observations of street segments. This simultaneously maximizes sensitivity and recency that are lost through using Google Street View and reduces privacy concerns and expenses involved with using remote sensing and drones.

## Methods

Our objective was to construct and refine a sampling frame within the context of a depopulating urban setting using systematic observations of street segments. We sought a random population sample of households, and to achieve this we intentionally oversampled North Flint because this is the area of the city with the most vacancy, the highest transient population, the most severe disinvestment over time (largely in the form of white flight), a negative stigma regionally, and is the focus of a local foundation’s grant strategy [2, 4, 8, 12, 13].

**Fig. 1:**
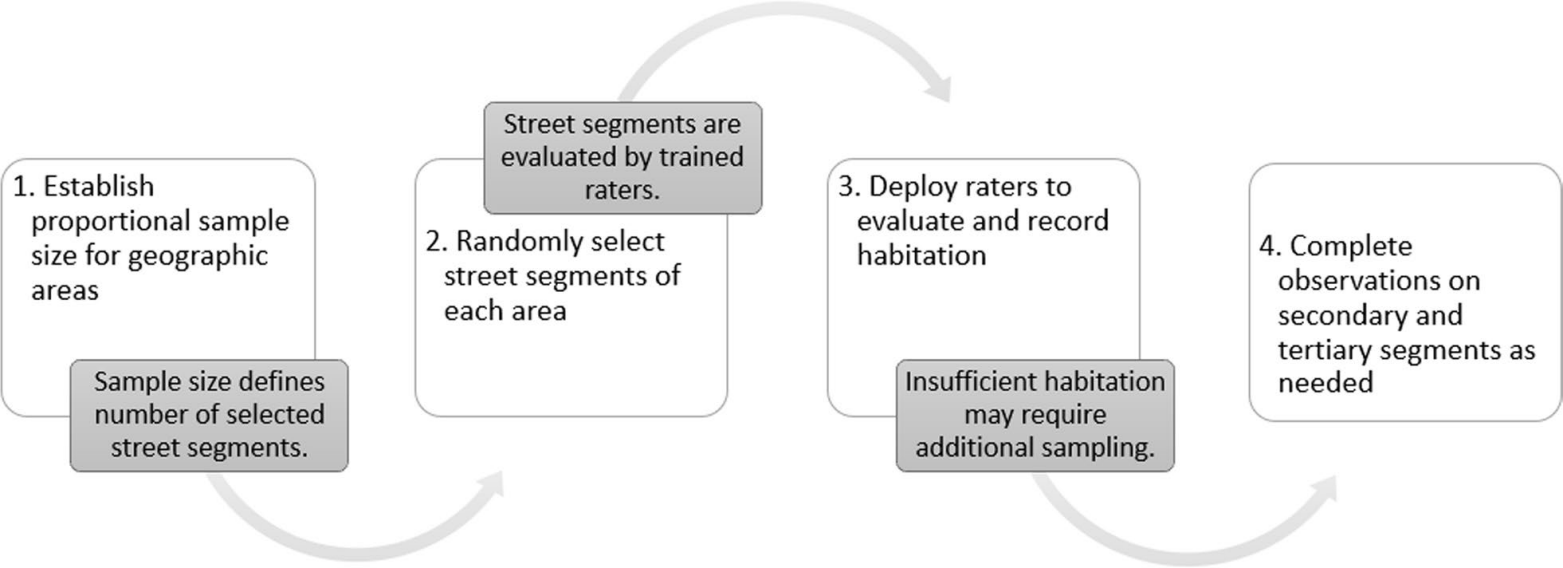
Summary of four steps involved the PROP method

We devised a four-step population randomization observation process (PROP), summarized in Fig. 1. For step one, we used the most recent census total population data (2010) to establish the necessary sample size for a population health study, for each area. To achieve a 1% sample of the total population of Flint we determined that a random sample of 350 households for the entire city, together with an additional 50 households in North Flint, was appropriate for this study. The additional 50 households were added in North Flint to achieve sufficient statistical power to detect differences between North and South Flint. We determined that out of 400 total households surveyed across Flint, each with an average of 2.5 people per household, our sample would yield ~ 1000 residents. We used a two-tiered approach, utilizing census tract and census block group (CBG) population estimates. First, we took the proportion of the population per census tract (Census Tract Population/Total Flint Population) and multiplied it by 350 and rounded to the nearest whole integer. The sum for all census tracts needed to equal 350 and required slight adjustment due to rounding. We took the proportion of census block group population (CBG Population/CT Population) and multiplied by the integer from the previous step, rounded again, to ensure the total equaled 350 again. This resulted in the desired sample size of 350, to which we would add an oversample of 50 households in North Flint.

**Fig. 2:**
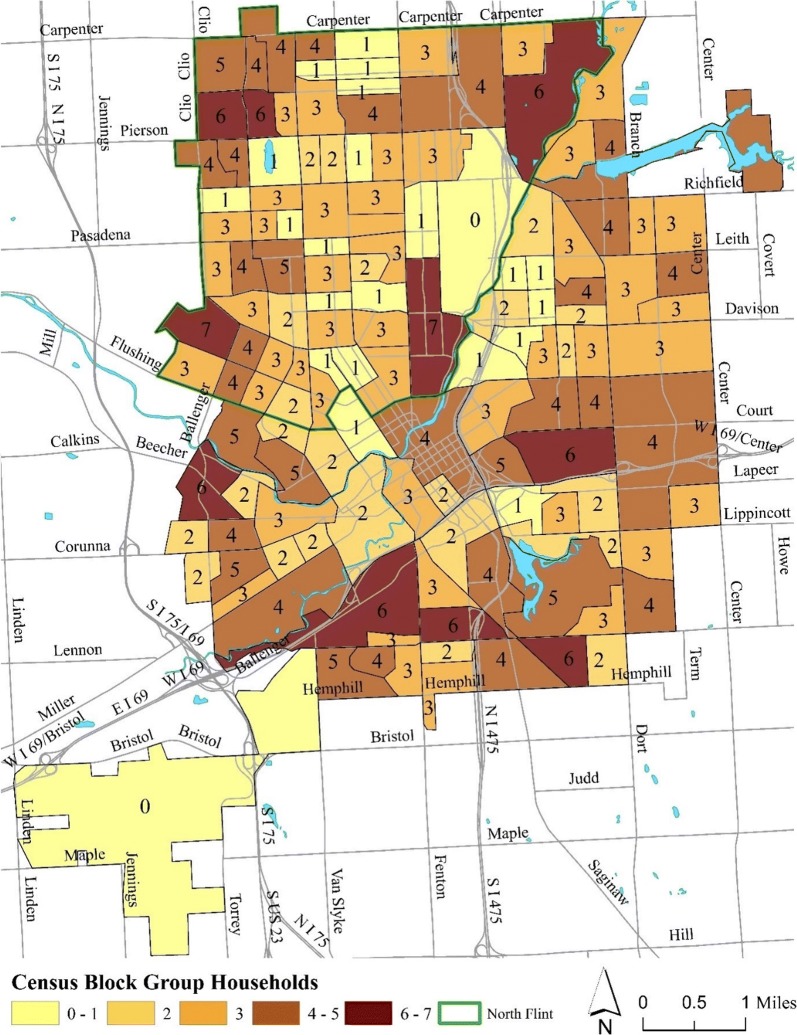
Flint, Michigan segmented by Census Block Groups, with no. households needed for population random sample (FCHES Methodology Core)

To create the oversample for North Flint (defined as the area north of Flushing Rd and west of the Flint River, creating the dividing line indicated in green in Fig. 2), the same method was used to determine how many of the 50 additional samples would be assigned to each CBG. The total population in North Flint is 37,086 [18]. We took the proportion of the census tract population for just North Flint (CT Pop/37,086), multiplied it by 50, rounded to the nearest whole integer, and ensured the sum was 50. Next, we took the proportion of CBG population (CBG Pop/CT Pop), multiplied it by the integer from previous step, rounded and ensured the sum equaled 50 again. The 50 households in the North Flint were then added to the initial 350 within the CBGs. Figure 2 shows the CBGs as polygons with the associated sample size in each CBG displayed as a number within the polygon.

For the second step, we exported the spatial street segment data into Microsoft Excel and used the Random Function to randomly select 400 street segments across all CBGs (based on the algorithm in step one).

For step three, we deployed raters to conduct in-person observations for signs of habitation for all dwellings on the randomly selected 400 street segments. All dwelling types, including single-family homes, apartment complexes, trailer parks, and retirement communities were treated similarly—if the dwelling was on a selected street segment and it was visible from the curbside, raters sought signs of habitation. Because raters were not permitted to enter private property, they could not attempt to assess habitation in individual apartment units. The (eight) Flint resident raters were selected because of their comfort and familiarity with the city. Raters utilized a form in EpiCollect v5 [1] programmed to document habitation on those randomly selected street segments. The form contained front- and end-matter questions designed to manage logistics. For example, the first question on the assessment was, “Who drove today?”, a question asked only to manage mileage reimbursement submission. Similarly, the last question on the assessment, “Did you feel safe on this street segment,” was used to determine which raters felt comfortable in all areas of the city. Besides these logistics-oriented questions, the form contained two questions of interest to the present study. The first, “Are there any inhabited dwellings?” determined whether raters received the second, “Are there at least 2 inhabited dwellings on this block?”

To complete the assessment, pairs of raters traveled together during daylight hours to the assigned starting address. They walked together (looking for signs of habitation in dwellings) on the street segment until they reached an intersecting street or dead end, turned 180°, then walked back to their starting point. They completed the EpiCollect form on electronic tablets while walking. The assessments were completed in January–March 2018. Raters were trained in a 1-h session that included orientation to EpiCollect, orientation to electronic tablet navigation, safety measures, procedures for answering questions from people on the street segments. The training also included guidance about how to look for signs of habitation (e.g., illumination, shoveled snow, children’s toys in the yard, people visible in the windows, or trash set out for collection). Raters were encouraged to use their subjective reasoning in assessing habitation. Raters were instructed to not discuss habitation while conducting assessments. A simultaneous testing approach was used and households were considered inhabited when at least one rater indicated such.

For the fourth step, if habitation on any randomly selected street segment in step three was ≤ 1 (i.e. only one household on the entire block appeared to be inhabited) we completed observations on a set of secondary, randomly selected street segments. We established the threshold of two or more inhabited households because our ultimate goal was conducting a population health survey. We assumed 50% of households might refuse. If the first street segment only had one inhabited household, a back-up would need to be on-hand in the case that residents in the sole inhabited household from the first street segment refused. Thus, when primary street segments had less than two, we randomly selected and observed back-up (secondary, tertiary, and so on) street segments within the CBGs until at least two occupied homes were observed on a street segment. Data were exported from EpiCollect and descriptive statistics were generated using R statistical software. We conducted a proportional odds (ordinal) logistic regression to determine how PROP assessment level co-varied with census-reported vacancy data for all CBGs in the city.

## Results

**Fig. 3:**
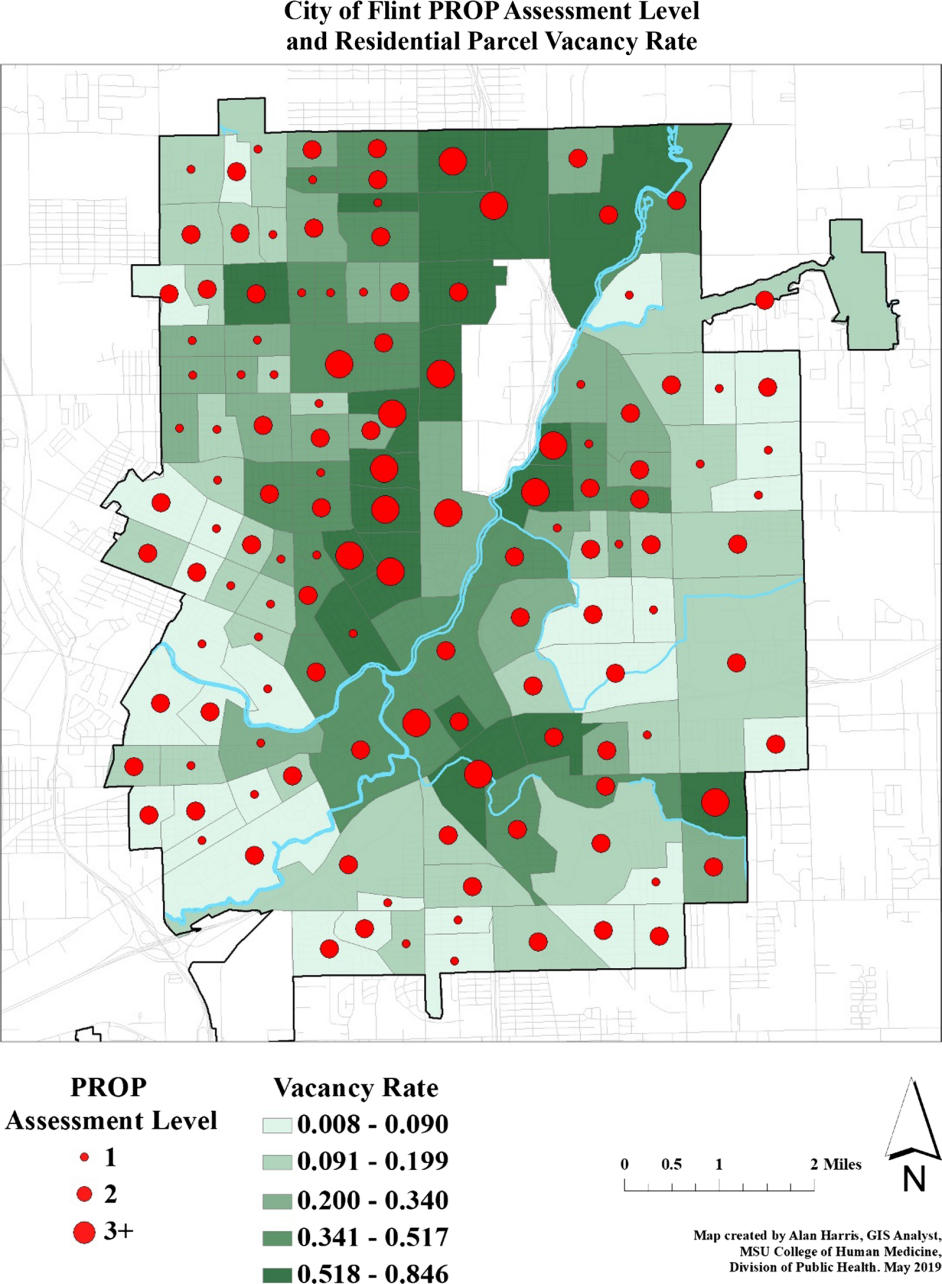
Flint, Michigan Census Block Group segments shaded by vacancy with spot size indicating number of PROP assessments needed

On average, raters spent approximately 2 min assessing habitation for all households on a given street segment. For 71.5% of the 400 primary street segments, raters had agreement (i.e., both raters deemed the house either inhabited or uninhabited). Among 400 randomly selected street segments, 130 (32.5%) required assessment of secondary street segments due to habitation below our established threshold. Among these 130 primary street segments, 28 (21.5%) required assessment of tertiary (or more) street segments. At the CBG level, 67 of 131 (51%) CBGs had at least one street segment that needed a secondary back-up assessment, and 17 of 131 CBGs (13%) had at least one street segment that needed tertiary or more back-up assessments. The level of assessment required (primary, secondary, and tertiary+) was designated the PROP assessment level and is displayed with red circles in Fig. 3. Ordinal logistic regression results estimated that every 10% increase in vacancy in a CBG made it 57% more likely that we would need to move on to complete PROP assessment for a second (or if they were already looking at a second, for a third) street segment.

## Discussion

Our PROP results indicate that locating inhabited homes for a population sample in a depopulating city like Flint has major challenges—the primary randomly selected street segments were sufficient in only 31% of CBGs in Flint. Results also indicate that there was utility in having Flint residents trained as raters. We believe that harnessing residents’ existing comfort with traversing low occupancy neighborhoods and allowing them to collect real-time habitation data holds significant advantages over other widely used methods (e.g. relying on aged municipal parcel vacancy data or using Google Earth observations). Houses that data collectors indicated were inhabited could have easily been considered as “uninhabited” via other methods. This could cause residents of ambiguous homes (likely to be the most marginalized residents with unmet health needs) to be overlooked.

For public health, monitoring public health outcomes and inequalities requires representative local- and community-level public health data [17]. To employ the PROP method in other areas for health research, we recommend this novel four-step process: (1) Obtain recent total population data for well-defined community areas and determine total sample size, (2) Randomly select street segments within each of the community areas; (3) Deploy raters to conduct observations to identify habitation within the randomly selected segments; and (4) Complete observations for secondary and tertiary segments as needed.

Using the systematic observations approach has several inherent strengths and weaknesses. Having raters canvass a geographic region on foot maximized sensitivity. Although raters spent very little time on each street segment they could use all senses to inform reasoning about whether households on the street segment were inhabited (e.g., visual, auditory, olfactory). The total number hours spent doing PROP assessments was approximately 94, counting driving and assessment time for each block. In a city with higher housing density, such that each street segment has many more homes to assess for habitation, this process would have taken more time. However, while the observation process requires more hours of observation up front, this method has the potential to reduce future work not achievable through other methods (e.g., aerial imagery). By identifying inhabited homes to define the sample, spatial data on occupied/vacant homes can be updated and used for future data collections including in-person surveys, postcard mailings, etc. If surveys were attempted in person or postcards mailed prior to employing the PROP method, many hours and study expenses would be spent needlessly in unoccupied street segments.

The ethics, reliability, and expense issues discussed in our introduction had us develop the PROP method as supplement/alternative to assessment done with images. A major limitation of using Google Street View imagery is the frequency with which imagery is collected [15]. In a rapidly changing neighborhood images are likely not reliable due to the rapid changes in housing in such settings. For spaces where habitation is clearly discernable and reliable via imagery, and where using or acquiring images is ethical and cost-effective, virtual image might be best.

This method has utility for settings beyond Michigan and the United States, as establishing epidemiologic samples in non-urban areas with high vacancy and transiency (e.g. areas used by dispersed nomadic populations) is particularly challenging where census data do not exist [11]. In such settings, the PROP method might be combined with remote spatial techniques to establish household-level epidemiologic population samples. Other studies employing the PROP assessment method could choose to use either a CT-based or a CBG-based sampling system instead of using the two-tiered sampling system. Regardless of the approach, close evaluation of sample changes (i.e. losses or gains to the sample in each geographic area), and consideration of the impacts of those changes, are required.

## Conclusion

In our study, using the PROP was an important step to ensure a representative population sample. Although census data were critical as a starting point for understanding which geographic areas might be inhabited, they were not recent enough to be reliable or to understand geographic variation in occupancy within census units. The PROP method has utility for any setting with recent (within 5 years) estimates of total population within well-defined areas. This method is also useful for establishing samples in areas with high vacancy due to other reasons (e.g. high foreclosure or natural disasters). If needed, during the PROP direct observation phase, other data collection such as a pollution sampling, neighborhood audit, or collection of 360 imagery could be carried to further increase efficiency.

This method offers a replicable systematic observation process for refining a random population sampling frame within the context of a depopulating urban setting. The results of this approach allowed for efficient recruitment efforts during our subsequent population health study and in-depth familiarity with sampled street segments across the city. Future studies using this method might also consider using PROP data to substantiate the validity of existing municipal vacancy data.

## Data Availability

The datasets generated and/or analyzed during the current study can be made available by request to flintareastudy@msu.edu.

## Abbreviations

ACS: American Community Survey
CBG: census block group
CT: census tract
EVD: Ebola virus disease
Pop: population
PROP: population randomization observation process
SSO: systematic social observation
